# Exploring Challenging Properties of Liquid Metallic Systems through Machine Learning: Liquid La and Li_4_Pb Systems

**DOI:** 10.1021/acs.jctc.4c00049

**Published:** 2024-04-01

**Authors:** Beatriz G. del Rio, Luis E. González

**Affiliations:** Departamento de Física Teórica Atómica y Óptica, Universidad de Valladolid, 47011 Valladolid, Spain

## Abstract

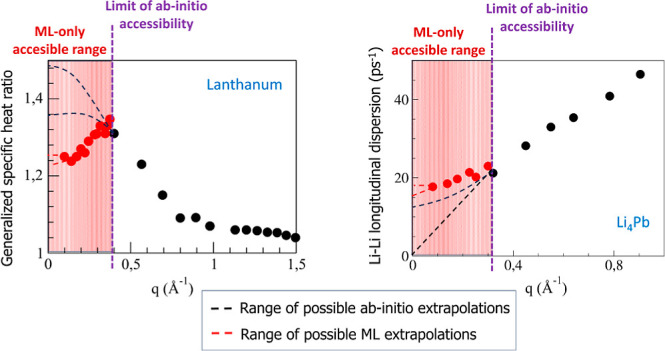

In this machine learning (ML) study, we delved into the unique properties of liquid lanthanum and the Li_4_Pb alloy, revealing some unexpected features and also firmly establishing some of the debated characteristics. Leveraging interatomic potentials derived from ab initio calculations, our investigation achieved a level of precision comparable to first-principles methods while at the same time entering the hydrodynamic regime. We compared the structure factors and pair distribution functions to experimental data and unearthed distinctive collective excitations with intriguing features. Liquid lanthanum unveiled two transverse collective excitation branches, each closely tied to specific peaks in the velocity autocorrelation function spectrum. Furthermore, the analysis of the generalized specific heat ratio in the hydrodynamic regime investigated with the ML molecular dynamics simulations uncovered a peculiar behavior, impossible to discern with only ab initio simulations. Liquid Li_4_Pb, on the other hand, challenged existing claims by showcasing a rich array of branches in its longitudinal dispersion relation, including a high-frequency LiLi mode with a nonhydrodynamic optical character that maintains a finite value as *q* → 0. Additionally, we conducted an in-depth analysis of various transport coefficients, expanding our understanding of these liquid metallic systems. In summary, our ML approach yielded precise results, offering new and captivating insights into the structural and dynamic aspects of these materials.

## 1 Introduction

Computer simulation techniques play an important role in providing new insights into many problems concerning condensed matter physics and materials science as well as an additional explanation and confirmation of experimental results.

Starting with the groundbreaking work of Alder and Wainwright^1^ on the fluid to solid phase transition of a model hard sphere system, the molecular dynamics (MD) simulation technique has undergone a huge development. The next step into this process was to account for the interactions beyond simple elastic collisions so as to provide a more realistic description of the dynamic behavior of atoms and/or molecules. This was achieved by Rahman^2^ who implemented a classical molecular dynamics (CMD) simulation, where the classical equations of motion were solved for a realistic model of a physical system, made up of real (argon) atoms interacting via a Lennard-Jones-type pair potential. Subsequently, this CMD approach was extended to more complex systems by introducing more elaborated types of interatomic potentials intended to describe the physics behind the interactions among the atoms. Nevertheless, in certain cases, such as systems where the electronic and atomic structures are closely interwoven, relying solely on interatomic empirical potentials is insufficient. It becomes imperative to incorporate an explicit description of the electronic properties into the simulation.

A significant advancement was made by merging MD simulations with density functional theory (DFT).^3−5^ This breakthrough enabled the direct determination of the potential responsible for the interactions in a many-body system, starting directly from the electronic configuration of its components, thereby making possible the simulation of covalently bonded and metallic systems. This approach has proved to be so accurate that the ab initio molecular dynamics (AIMD) methods based on DFT have now become the usual technique for the study of a wide range of condensed matter systems. Most AIMD methods are based on the Kohn–Sham orbital representation of DFT; however, its application poses heavy computational demands and therefore AIMD simulations are severely limited by the finite system size (∼100 s of atoms) and short time scales (∼10 s of ps). However, there are some physical magnitudes whose accurate determination would involve the use of length and time scales which are not currently accessible by these AIMD simulation methods. For example, and within the realm of liquid systems, we mention the long wavelength limit of a range of static and time-dependent correlation functions as well as some transport coefficients. A proper account of these physical magnitudes requires the use of large number of atoms (tens of thousands) along with much longer simulation times (∼100–1000 s of ps), impossible nowadays with AIMD methods, but achievable by resorting to CMD simulations based on interatomic potentials at the cost of a significantly reduced accuracy.

Over the past decade, machine learning (ML) interatomic potentials have been developed as a way of addressing the abovementioned problems. By employing a database of quantum-mechanical (commonly DFT-based) calculations, it is possible to construct interatomic potentials that allow system sizes and simulation times typical of CMD simulations but with quantum mechanical accuracy.

We have chosen two distinct systems, liquid lanthanum (l-La) and liquid Li_4_Pb, to show the versatility and capability of ML potentials to study various aspects of liquid metal behavior inaccessible to AIMD.

Two basic magnitudes related to the structural short-range order in a liquid metal are the static structure factor, *S*(*q*) and the pair distribution function, *g*(*r*).

In the case of l-La near melting, the available experimental structural data were obtained more than 40 years ago by Breuil and Tourand^6^ by means of neutron diffraction (ND) and, a few years later, by Waseda and Tamaki^7^ through X-ray diffraction (XD). Since then, no additional measurements of *S*(*q*) have been reported for this metal. As for the thermophysical magnitudes of l-La, there are experimental data for the adiabatic velocity of sound^8^ and shear viscosity.^9^ On the theoretical side, few works have studied the structural properties of l-La, and among them, we mention the study by Waseda et al.^10^ who used effective interionic pair potentials, derived within the pseudopotential perturbation theory, combined with the Percus–Yevik integral equation to calculate static structure, i.e., *S*(*q*) and *g*(*r*), of some liquid rare-earth metals. More recently, Patel et al.^11−13^ used a pseudopotential combined with the hard sphere reference system to evaluate the *S*(*q*) and *g*(*r*) as well as some thermodynamic and electronic properties of the liquid lanthanide metals.

The Li_4_Pb liquid alloy has already attracted a great deal of both theoretical and experimental work. In common with other binary alloys with a large mass disparity between both components, it exhibits a high-frequency mode with an associated phase velocity much greater than the extended hydrodynamic sound as found in both MD studies^14−18^ and inelastic neutron scattering (INS) experiments.^19,20^ In fact, this high-frequency mode was first found in a CMD study of Li_4_Pb^14,15^ using interionic pair potentials modeled with a hard core repulsion plus a screened Coulombic interaction. These results showed that the high-frequency mode produced a high-frequency side peak appearing in the Li–Li partial dynamic structure factor. It was interpreted as an extended hydrodynamic mode with an associated phase velocity of ≈7500 m/s and was named the “fast sound” mode.

Additional CMD simulations using the same interionic interaction but with a larger number of particles^16^ confirmed the previous results and were able to discern two branches of collective excitations, as obtained from the maxima in the longitudinal current correlation spectra, that were identified as the fast sound mode and a new slow sound mode. It was concluded that these two modes merged, at very low *q*-values, into the usual hydrodynamic sound. Both modes showed a sharp transition in their phase velocities around *q*_c_ ≈ 0.08 Å^–1^, with an abrupt increase for *q* > *q*_c_ toward a “fast velocity” around 8000 m/s in the case of the high-frequency mode and a decrease toward a “slow velocity” around 1200 m/s in the case of the low frequency one. However, another study using CMD simulations in combination with the generalized collective modes (GCM) method^17^ found two branches of collective excitations which in the *q* → 0 limit were identified as a hydrodynamic (low-frequency) branch and an optic-like (high frequency) branch that tends to a nonzero frequency instead of merging with hydrodynamic sound. Similar conclusions were also obtained from CMD simulations analyzed within the viscoelastic model by Anento et al.^18^

On the other hand, the INS experiments,^19,20^ that were limited to *q* larger than 0.6 Å^–1^ due to kinematic conditions, confirmed the existence of this high-frequency mode but with an associated phase velocity around 4500 m/s. Moreover, they suggested that this branch had features that pointed to a kinetic (nonhydrodynamic) mode with a nonzero value in the *q* → 0 limit.

There are therefore two contradicting interpretations on the behavior of the high-frequency mode as the wavelength increases (either it merges with hydrodynamic sound or it behaves as a kinetic mode). The comparison with the experiment is not conclusive due to the wavevector region explored and moreover is somewhat hindered by the use of an effective potential in the CMD simulations that may not capture all the details of the true interactions among atoms in the alloy. Recent first-principles simulations with realistic interactions derived from electronic structure calculations^21,22^ appear to support the optic-like view, but in this case, the problem lies in the value of the smallest wavevector allowed by the periodic boundary conditions inherent to the calculations, which is 0.32 Å^–1^. This value is a direct consequence of the small number of particles affordable in such high cost simulations and is quite large so as to make conclusive statements on the behavior of the modes for *q* → 0.

This paper reports a simulation study of several static and dynamic properties of l-La at thermodynamic conditions just above melting at ambient pressure and the liquid Li_4_Pb alloy just above its liquidus temperature. The study has been performed first using AIMD simulations, then creating the corresponding ML interatomic potentials based on the aforementioned AIMD simulations, and finally using these ML potentials in CMD runs with much larger time and length scales.

An important aim of this study is to calculate, using ML simulation methods, the spectrum of collective excitations in the liquid Li_4_Pb alloy and analyze them, with special emphasis in its controversial high-frequency branch, and in particular its behavior in the long wavelength region. For l-La, we provide the first AIMD study and use the ML derived interatomic potential to better analyze the results in the hydrodynamic regime, uncovering an unconventional behavior in the generalized specific heat ratio.

The review is structured as follows: the next section summarizes the basic ideas underlying the AIMD and ML simulation methods along with some technical details. In Section 3, we report the results of the calculations for both l-La and the Li_4_Pb alloy, which are compared with the available experimental data along with some discussion. Finally, a brief summary and conclusions are given in Section 4.

## 2 Computational Method

The reference database of l-La was generated by AIMD simulations performed using the DFT-based QUANTUM ESPRESSO (QE) package.^23,24^ Within this framework, the electronic exchange-correlation energy was described by the generalized gradient approximation (GGA) of Perdew–Burke–Ernzerhof.^25^ The ion–electron interaction was accounted for by means of an ultrasoft pseudopotential generated from a scalar-relativistic calculation including nonlinear core corrections. In this pseudopotential, the 5s, 5p, 5d, 6s, and 6p orbitals are explicitly considered as valence states, and 11 valence electrons per atom were employed during simulations. The kinetic energy cutoff for the plane-wave expansion of the wave function was 530 eV, and the single Γ point was used for sampling the Brillouin zone.

For the liquid Li_4_Pb alloy, the reference database was obtained by AIMD simulations performed with the DFT-based VASP package.^26−29^ We used the projector augmented wave all-electron description of the electron–ion-core interaction provided by the VASP distribution, which considers one and four valence electrons for Li and Pb, respectively; moreover, the exchange correlation functional used was again GGA, while the kinetic energy cutoff for the plane-wave expansions was taken as 480 eV.

We have employed the SIMPLE-NN package^30^ to develop deep neural network potentials (DNNPs) which, due to their high flexibility, are a suitable model to represent the challenging problem of the potential energy surface of a liquid metal or alloy. To transform the atomic coordinates into atomic descriptors, SIMPLE-NN employs Behler–Parrinello Gaussian functions.^31,32^ For l-La, we used the Gastegger method^33^ to select the parameters of the 35 total Gaussian functions employed, whereas for Li_4_Pb, the Imbalzano method^34^ provided better parameters for the 36 Gaussian functions of each element.

The choice of either method (Gastegger or Imbalzano) is based on the better performance obtained when applying the learning process to the training data sets. The main difference between both methods lies in the expressions employed to select the widths and positions of the Gaussians. In particular, the Gastegger method uses a fixed width for the shifted Gaussians that is equal to the distance between the centers of the two adjacent functions, whereas the Imbalzano method increases the width as the distance increases, effectively creating a finer grid closer to the central atom, where small variations of the position can have a greater impact on the potential.

The neural network employed for the DNNP of l-La consisted of three layers, with 50 neurons per layer. The loss function included the energy per atom, atomic forces, and stress. The DNNP was trained on more than 11,700 configurations, using a training and validation split of 80:20.

In the case of the DNNP of liquid Li_4_Pb, the neural network architecture was composed of four layers with 50 neurons each. The training configurations included data from four different concentrations of the Li–Pb alloy: Li_0.17_Pb, Li_0.50_Pb, Li_0.62_Pb, and Li_0.80_Pb. We selected 200 configurations from each concentration, taken from a previous AIMD study,^21^ and the corresponding energies and forces were computed with VASP. However, due to extrapolation errors that appeared when testing the DNNP, additional AIMD runs were performed for liquid Li_0.17_Pb and liquid Li_0.80_Pb, maintaining the temperature but increasing the atomic density by a 20% and by a 40% so as to allow for configurations where shorter interatomic distances were present. From these new AIMD runs, a total of 190 additional configurations were included in the training of the final DNNP. Due to the small number of total configurations, a training and validation split of 90:10 was employed. The loss function included the energy per atom and the atomic forces.

While in principle, it would seem unnecessary to include additional compositions apart from the 80% Li in the training data set in order to develop the NN potential, in fact it turns out to be profitable to do so for two reasons: first because the instantaneous local atomic arrangements in the alloy can fluctuate departing from the nominal alloy concentration, especially when considering samples with a large number of atoms; and second, from a more practical viewpoint, because such strategy will allow the future study of the alloy at different concentrations using the same DNNP.

Note that the inclusion of the stress in the loss function for l-La, as well as the larger number of configurations included in the data set for that system, as compared to the case of Li_4_Pb, is justified by the higher degree of complexity of the interactions in this system, as evinced by the much higher number of valence electrons needed in the AIMD study in order to produce accurate forces and energies.

Both DNNPs employed fully connected layers, with the hyperbolic tangent as the activation function, were trained using a mini-batch of 30, used random sampling along with Adam optimizer with a learning rate of 0.001, and employed an *L*^2^ regularization of 10^–6^. The mean-squared-error was employed as the objective function during all training. The coefficients for various quantities when included in the loss function were as follows: 1.0 for the energy per atom, 0.1 for the atomic forces, and 10^–4^ for the pressure. Once the DNNPs were trained, they were utilized in LAMMPS^35^ to perform the large-scale MD simulations.

Table 1 provides details of the different kinds of simulations performed for the two systems considered in the study. In all cases, the simulation samples with *N* particles were enclosed inside a periodic cubic cell with a size determined by the experimental ionic number density. After thermal equilibration, the number of equilibrium configurations, *N*_c_, given in Table 1 was subsequently used for the evaluation of the static and dynamic properties of both systems.

**Table 1:** Input Data for the Systems Studied in This Work^a^

	La		Li_4_Pb	
code	QE	ML	VASP	ML
*N*	100	6400	330	21,120
ρ (Å^–^^3^)	0.0258	0.0258	0.0436	0.0436
*T* (K)	1250	1250	1075	1075
*N*_c_	13,200	180,000	10,500	100,000
Δ*t* (fs)	6.0	1.0	4.0	4.0

a ρ is the total ionic number density, *T* is the temperature, *N* is the number of particles in the simulation cell, Δ*t* is the ionic timestep, and *N*_c_ is the total number of configurations.

## 3 Results and Discussion

### 3.1 Static Properties

#### 3.1.1 Liquid La

**Figure 1:**
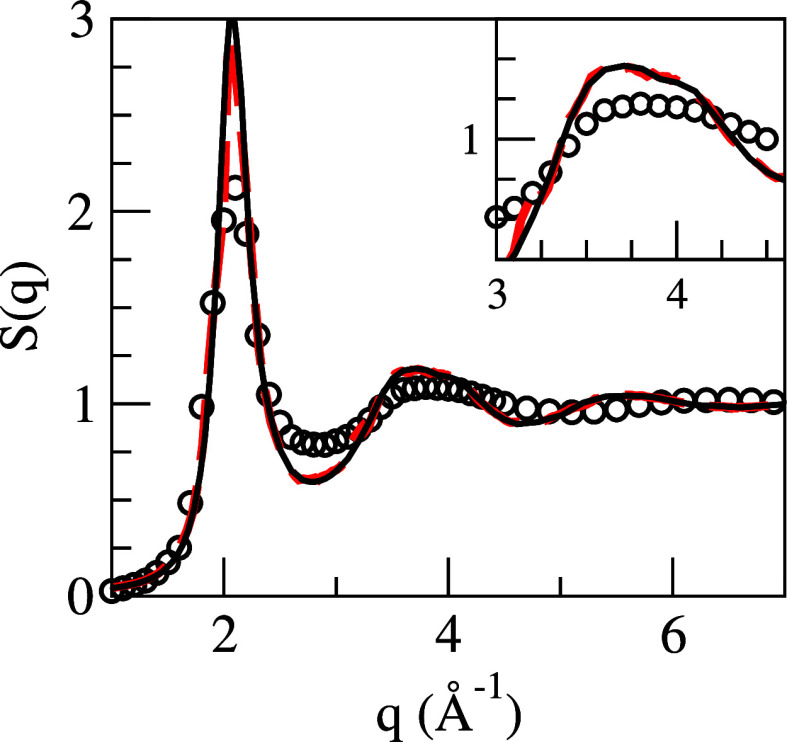
Static structure factor, *S*(*q*), of l-La. Lines: present AIMD (dashed red) and ML (continuous black) calculations; open circles: XD data from ref (7). The inset shows a closer view of the second maximum.

Figure 1 depicts the calculated static structure factor, *S*(*q*) as obtained from the AIMD calculation along with the ML result and the XD data of Waseda et al.^7^ Notice that the AIMD and ML results are practically identical, and the comparison with the XD data shows agreement for the position of the main peak, *q*_p_ = 2.1 Å^–1^, although the calculated amplitudes are more marked; moreover, both simulations predict an asymmetric shape of the second peak of the *S*(*q*) which is not found as pronounced in the XD data. Nevertheless, we notice that this feature has been experimentally observed in several liquid metals^36−38^ and has been related to the presence of a substantial amount of icosahedral order in the liquid.

Concerning the evaluation of *q*-dependent magnitudes, the use of ML interatomic potentials allows us to reach considerably smaller *q*-vectors, allowing for a more detailed investigation into the long-wavelength (*q* → 0) region. The smallest attainable value by the ML method was *q* = 0.099 Å^–1^, whereas in the case of the AIMD calculation, the smallest value was *q* = 0.400 Å^–1^. We have exploited this feature of the ML method to estimate the isothermal compressibility, κ_*T*_, by resorting to the relation *S*(*q* → 0) = ρ*k*_B_*T*κ_*T*_, where *k*_B_ is Boltzmann’s constant. First, the ML-calculated *S*(*q*) has been extrapolated to *q* → 0 by a least-squares fit, *S*(*q*) = *s*_0_ + *s*_2_*q*^2^, of the *q*-values for *q* ≤ 1.0 Å^–1^. It yields a value of *S*(*q* → 0) = 0.0160 ± 0.001, which gave κ_*T*_ = 3.60 ± 0.20 (in units of 10^11^ m^2^ N^–1^). Other evaluations for this magnitude at the melting point have been proposed by some authors; thus, McAlister et al.^8^ estimated a value of κ_*T*_ = 4.24 from speed of sound measurements, while Blairs^39,40^ suggested a value κ_*T*_ = 4.30 obtained by using an empirical formula connecting isothermal compressibility, surface tension, and the Ornstein–Zernike correlation length. However, no direct experimental measurement is available to date.

**Figure 2:**
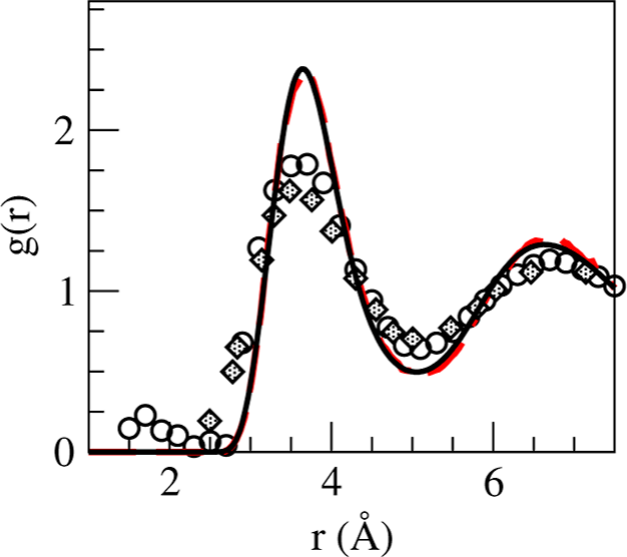
Pair distribution function, *g*(*r*), of l-La. Lines: present AIMD (dashed red) and ML (continuous black) calculations; open circles: XD data from ref (7); and full diamonds: ND data for l-La at 1250 K from ref (6).

Figure 2 shows the pair distribution function, *g*(*r*), which provides some insights into the short-range order in the liquid. Both AIMD and ML results are depicted along with the available experimental data.^6,7^ There is a fair agreement concerning the position of the oscillations, but the calculated amplitudes are greater with both AIMD and ML.

The average number of nearest neighbors (also called the coordination number, CN) around any given ion is evaluated by integrating the radial distribution function, 4πρ*r*^2^*g*(*r*), up to the position of its first minimum, *r*_min_ ≈ 4.97 Å. The calculated CN with AIMD and ML is ≈13.0, similar to those obtained for other simple liquid metals near melting.^41^

The common neighbor analysis^42−44^ (CNA) method provides additional insights into the short-range order as it gives three-dimensional information about the ions surrounding each pair of ions which are near neighbors. Each pair is characterized by four indices which allow us to discern among different local structures like fcc, hcp, bcc, and icosahedral environments. The present calculations (both AIMD and ML) show that the five-fold symmetry dominates in l-La because the sum of perfect and distorted icosahedral structures reaches a value of ≈53% of the pairs with the number of perfect ones being around 30%. The amount of local bcc-type pairs is also significant, ≈27%; however, there is virtually no vestige of fcc and hcp-type pairs.

#### 3.1.2 Liquid Li_4_Pb Alloy

**Figure 3:**
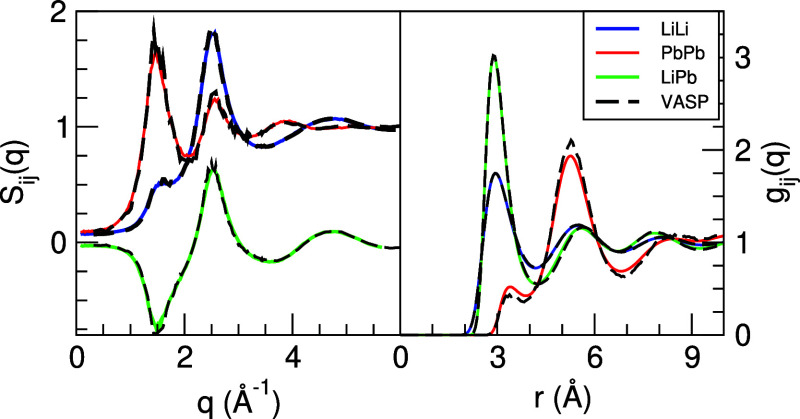
Ashcroft–Langreth (AL) partial pair distribution functions and static structure factors *S*_*ij*_(*q*) for the liquid Li_4_Pb alloy at 1075 K. Full blue, red, and green lines are the ML results, whereas dashed lines are the AIMD results.

Figure 3 depicts the ML calculated partial pair distribution functions *g*_*ij*_(*r*) and partial static structure factors *S*_*ij*_(*q*) along with the corresponding AIMD results. Both sets of results are practically identical, with very minor discrepancies concerning the amplitudes of the *g*_PbPb_(*r*).

We have evaluated the isothermal compressibility in the alloy by using the expression^45^
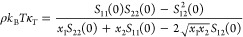
1where ρ is the number density of the alloy, *x*_*i*_ is the concentration of component *i*, and *S*_*ij*_(0) are the *q* → 0 values of the partials. These latter values were derived by extrapolating the ML-calculated *S*_*ij*_(*q*) in the same way that was performed for l-La. Now the smallest wavevector attained was *q* = 0.080 Å^–1^. Thus, we obtained (with an uncertainty ≈±0.001) *S*_LiLi_(0) = 0.0715, *S*_LiPb_(0) = −0.0275 and *S*_PbPb_(0) = 0.0920. The application of eq 1 yielded a value of κ_*T*_ = 8.18 ± 0.20 (in 10^11^ m^2^ N^–1^ units). There are no experimental data for this magnitude, but we will later compare it with the data for the adiabatic compressibility. Finally, we note that information about the ordering tendencies in the alloy is given by the long-wavelength limit of the Bhatia–Thornton (BT) concentration–concentration partial structure factor, *S*_cc_(*q*), which can be evaluated in terms of the previous partials.^45^ For this alloy, we have obtained *S*_cc_(*q* → 0)/(*x*_1_*x*_2_) = 0.0975, which clearly indicates a strong heterocoordinating tendency in the liquid Li_4_Pb alloy.

### 3.2 Collective Dynamics

#### 3.2.1 Liquid La

The collective dynamics of density fluctuations in a liquid can be described by the intermediate scattering function, *F*(*q*, *t*), which is the autocorrelation function of the microscopic *q*-dependent number density.^41^ Its time Fourier transform (FT) gives the frequency spectrum known as the dynamic structure factor, *S*(*q*, ω).

**Figure 4:**
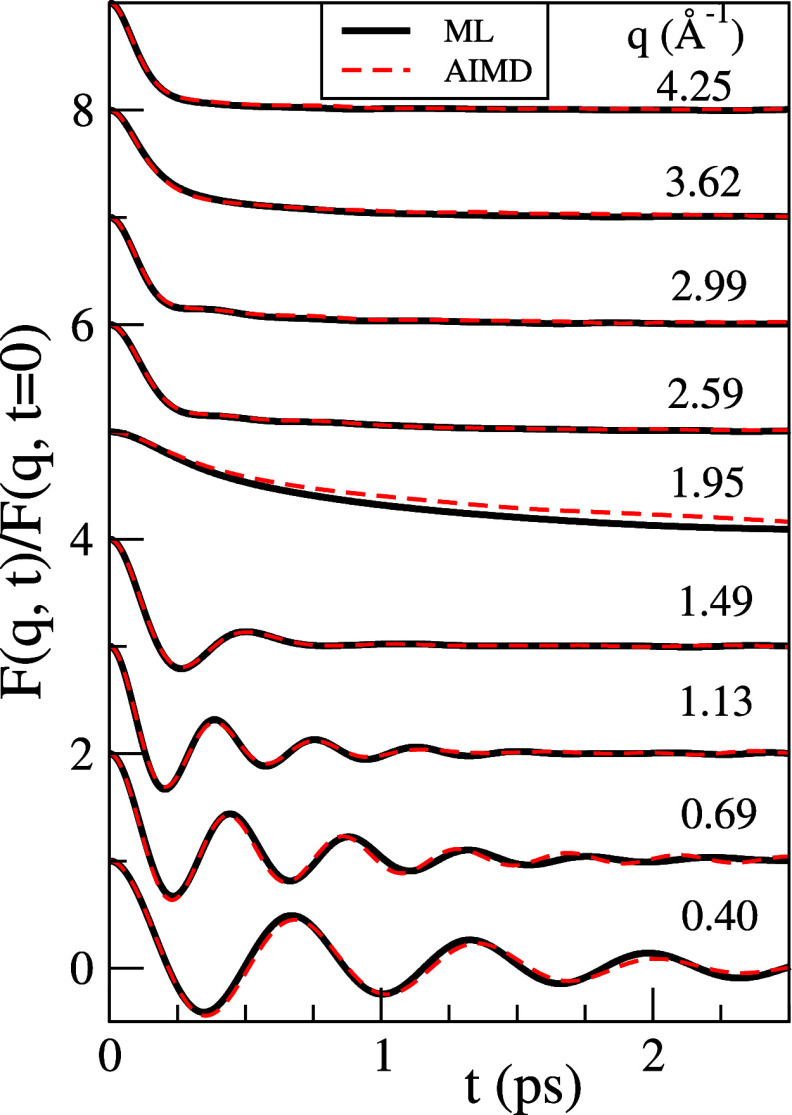
Normalized intermediate scattering function, *F*(*q*, *t*)/*F*(*q*, *t* = 0), of l-La at *T* = 1250 K for several *q* values.

Figure 4 shows, for several *q*-values, the AIMD results for *F*(*q*, *t*) along with the corresponding ML results. The calculated *F*(*q*, *t*) show a structure qualitatively similar to other liquid metals: at small *q*’s, there is an oscillatory form, suggesting wave propagation, which is combined with a decaying term that indicates the existence of relaxation modes. The interplay between these two terms evolves with increasing *q*-values with the decaying term smoothly and progressively overcoming the propagating mode, leading at *q* ≈ *q*_p_ to a very slow monotonic decay of *F*(*q*, *t*).

Further insights into the physical causes behind the propagation and relaxation mechanisms can be obtained from theoretical models where the *F*(*q*, *t*) is expressed using the formalism of memory functions,^41^ more specifically its second-order memory function *N*(*q*, *t*), i.e.
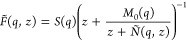
2where *F̃*(*q*, *z*) and *Ñ*(*q*, *z*) are the Laplace transforms of *F*(*q*, *t*) and *N*(*q*, *t*), respectively, *M*_0_(*q*) = *k*_B_*Tq*^2^/(*mS*(*q*)), and *m* is the atomic mass. The *N*(*q*, *t*) can be modeled by an analytical function with two exponentially decaying terms (slow and fast ones), i.e.
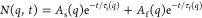
3where τ_s_(*q*) and τ_f_(*q*) represent slow and fast relaxation times, respectively. Physically, one term has a thermal origin, whereas the other is associated to the viscoelastic behavior of the liquid.^46−48^

We used *F*(*q*, *t*) obtained by the AIMD and ML simulations in order to evaluate the associated functions *N*(*q*, *t*). Then, they were fitted to eq 3 and were analyzed to find out whether they are consistent with a generalized hydrodynamic model (fast viscoelastic mode and a slow thermal one) or a generalized viscoelastic model, where the fast term is the thermal one. Specifically, we evaluated the generalized heat capacity ratio, γ(*q*), which in the *q* → 0 limit leads to the thermophysical value γ_0_, i.e., the ratio between the specific heats at constant pressure and constant volume.

If the slow mode is associated to the thermal relaxation, then *A*_s_(*q*) = (γ(*q*) – 1)*M*_0_(*q*), but if it is connected to the viscoelastic relaxation, then *A*_s_(*q*) = ω_L_^2^(*q*) – γ(*q*)*M*_0_(*q*),^47,48^ where ω_L_^2^(*q*) is the second frequency moment of the longitudinal current correlation function (see below), which is obtained directly from the simulations.

By exploring both possibilities, we have calculated the functions γ_th_(*q*) and γ_v_(*q*), which represent the values obtained for γ(*q*) when either the thermal or the viscoelastic relaxations proceed by the slow mode, respectively.

**Figure 5:**
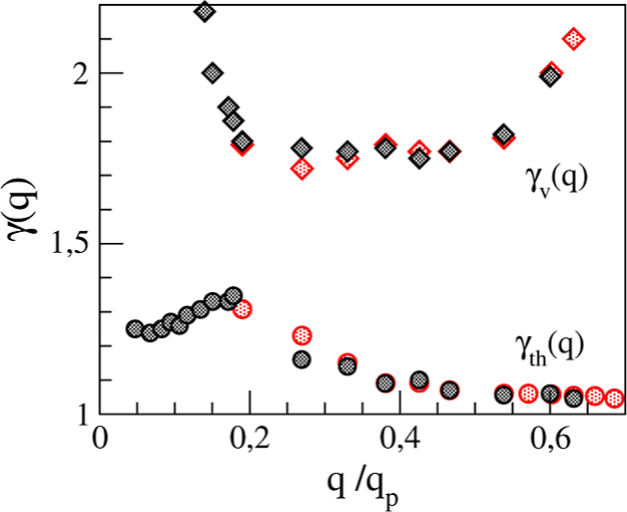
Generalized specific heat ratio, γ(*q*), as obtained from the generalized hydrodynamic model (circles) and the generalized viscoelastic model (lozenges). The red and gray symbols are the AIMD and ML simulation results, respectively.

The results are depicted in Figure 5, which shows the obtained γ_th_(*q*) and γ_v_(*q*) values within the range *q*/*q*_p_ ≤ 0.70 Å^–1^. Both functions exhibit a very different conformation, with γ_v_(*q*) taking values which are always greater than those of γ_th_(*q*); moreover, when *q* → 0, the γ_v_(*q*) becomes unphysically large. As for the γ_th_(*q*), we observe that the AIMD results show a monotonous increasing behavior down to the smallest attainable *q*-value (*q*/*q*_p_ = 0.19 Å^–1^); however, the ML values show that a noticeable change around that same *q*-value and the associated γ_th_(*q*) displays a slowly decreasing behavior toward *q* = 0, with a γ_th_(*q* → 0) ≡ γ_0_ ≈ 1.25.

We are not aware of any experimental data for the γ(*q*) of l-La but we note that the qualitative structure of the ML calculated γ_th_(*q*) is very similar to that reported in the experimental data of Hosokawa et al.^49^ for the γ(*q*) of l-Fe near melting. Specifically, the γ_exp,Fe_(*q*) begins with a value of ≈1.40 at *q*/*q*_p,Fe_ ≈ 0.07 Å^–1^ and smoothly increases reaching a value of ≈1.55 at *q*/*q*_p,Fe_ ≈ 0.21 Å^–1^ and then decreases toward a value of ≈1.1 at *q*/*q*_p,Fe_ ≈ 0.67 Å^–1^ (where *q*_p,Fe_ ≈ 2.98 Å^–1^).

Although there are no experimental data for γ(*q* → 0) ≡ γ_0_ of l-La near melting, we can compare it with two estimates, namely, γ_0_ = 1.23^39,40^ and 1.31,^50^ which were obtained by combining the experimental data of the adiabatic compressibility and the calculated isothermal compressibility.

The previous results suggest that the slow mode is associated with the thermal relaxation, and therefore, the generalized hydrodynamic model seems to be the appropriate one for describing the microscopic dynamics of l-La; moreover, it predicts a behavior for γ(*q*) which is qualitatively consistent with the experimental data for l-Fe and, furthermore, it also provides an estimate for γ_0_ that compares well with some semiempirical values.

**Figure 6:**
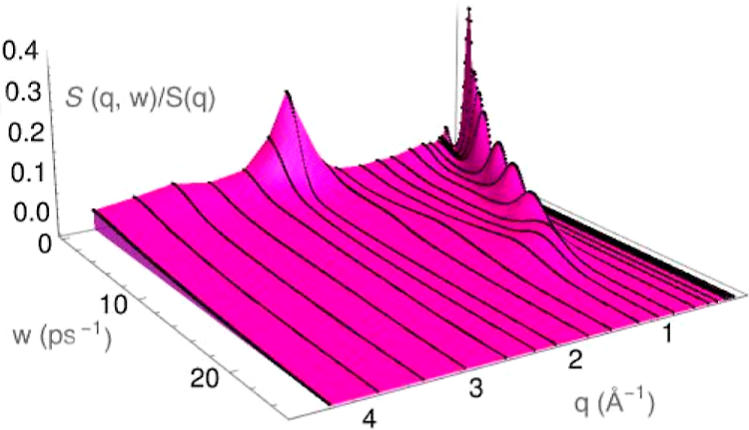
ML results for the dynamic structure factors, *S*(*q*, ω)/*S*(*q*), of l-La at *T* = 1250 K and several *q* values.

The dynamic structure factors, *S*(*q*, ω), are plotted in Figure 6, and show side peaks up to *q* ≈ (3/5)*q*_p_, whereas for greater *q*-values, we observe a monotonic decreasing behavior.

**Figure 7:**
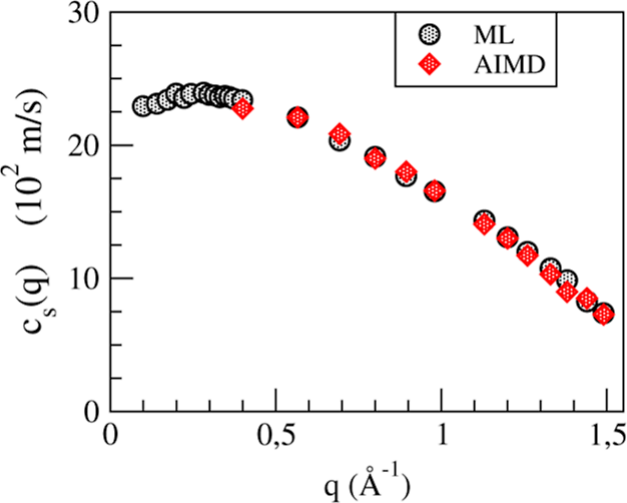
*q*-dependent adiabatic sound velocity of l-La at *T* = 1250 K. The red and gray symbols are the AIMD and ML simulation results, respectively.

We have determined the frequency of the side peaks as a function of the wavevector, namely, the function ω_m_(*q*), which represents the associated dispersion relation of the density excitations; therefrom, a *q*-dependent adiabatic sound velocity *c*_s_(*q*) = ω_m_(*q*)/*q* is obtained and has been depicted in Figure 7. Again, the AIMD and ML simulation results are practically identical but only the latter ones are able to clearly indicate the existence of positive dispersion. In the *c*_s_(*q* → 0) limit, this magnitude reduces to the bulk adiabatic sound velocity, *c*_s_, and by extrapolating the previous ML simulation results, we have obtained an estimate *c*_s_ = 2230 (±150) m/s which qualitatively agrees with the experimental data *c*_s,exp_ = 2022 m/s.^8^

Another important magnitude in the collective dynamics is the current due to the overall motion of the particles, **j**(*q*, *t*)
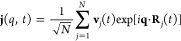
4where *N* is the total number of particles and **v**_*j*_(*t*) is the velocity of particle *j* at time *t*.^41^ From its longitudinal, **j**^*L*^(**q**, *t*), and transverse, **j**^*T*^(**q**, *t*), components, we have evaluated the associated longitudinal and transverse current correlation functions
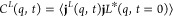
5
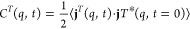
6and their respective time FT, the associated spectra *C*^*L*^(*q*, ω) and *C*^*T*^(*q*, ω).

**Figure 8:**
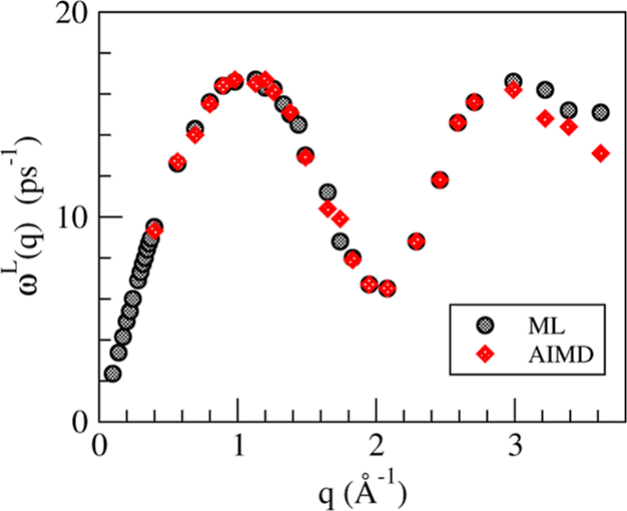
Longitudinal dispersion relation of l-La, as obtained from the maxima in the spectra of the longitudinal current, *C*^*L*^(*q*, ω).

For any fixed *q*-value, when the *C*^*L*^(*q*, ω) is plotted as a function of ω, we observe a maximum, and its associated frequency, namely, ω^*L*^(*q*), stands for the dispersion relation of the longitudinal modes. These are plotted in Figure 8 where it is observed that the AIMD and ML simulation results are very similar, although the ML approach again provides closer insights into the small *q*-region.

**Figure 9:**
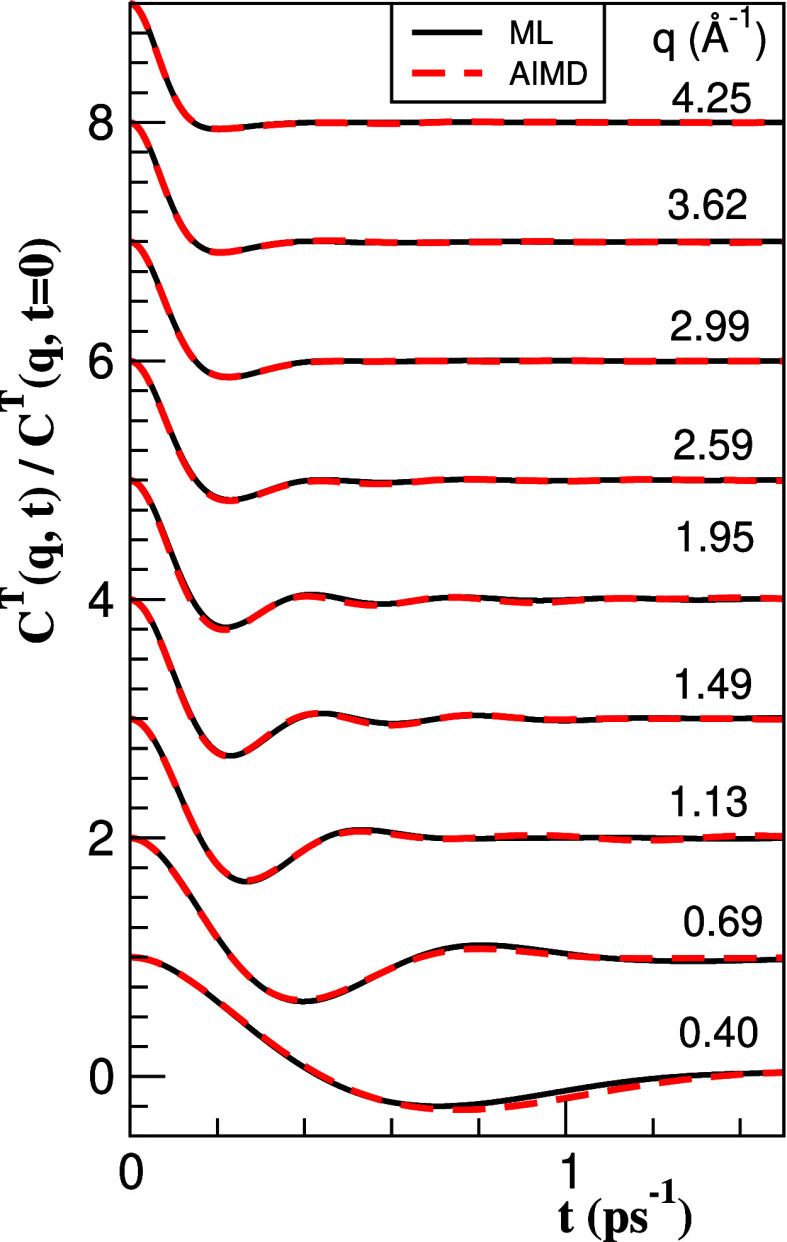
Normalized transverse current correlation function, *C*^*T*^(*q*, *t*)/*C*^*T*^(*q*, *t* = 0), of l-La at *T* = 1250 K for several *q* values.

Figure 9 depicts, for a range of *q*-values, the calculated AIMD and ML results for *C*^*T*^(*q*, *t*). For both small and large *q*, this function must be monotonically decreasing with time, but for intermediate *q* values, it shows oscillations.

Correspondingly, its spectrum, *C*^*T*^(*q*, ω), when plotted as a function of ω, shows within some *q*-range clear peaks that are related to propagating shear waves. According to the present ML results, the peaks start at *q* ≈ 0.15 Å^–1^ and last until *q* ≈ 3.0*q*_p_ when they fade away. Moreover, we have found a second, higher frequency peak that shows up over a much smaller *q*-range.

**Figure 10:**
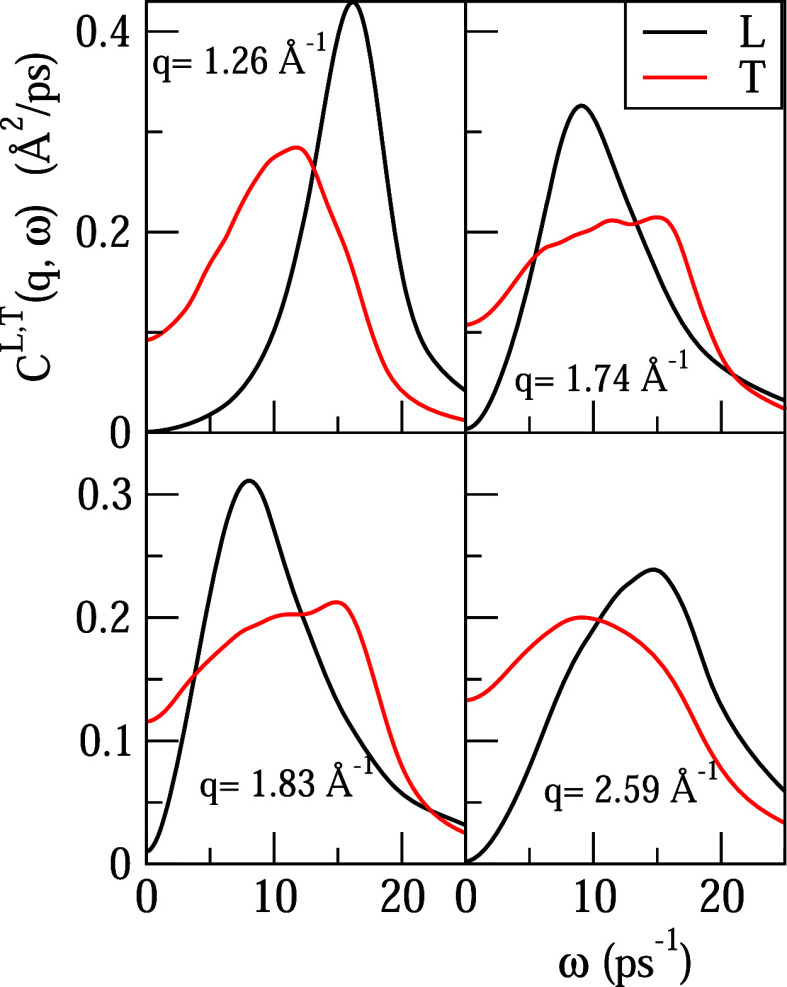
ML calculated spectral functions of the normalized longitudinal and transverse current correlation function, *C*^*L*^(*q*, ω) and *C*^*T*^(*q*, ω), respectively, for several *q* values.

Some examples of the behavior of *C*^*T*^(*q*, ω), along with the corresponding longitudinal spectrum, *C*^*L*^(*q*, ω), are shown in Figure 10, where the appearance and later disappearance of the high-frequency peak for increasing *q* is observed. Moreover, it is also seen that the peak in the longitudinal spectrum at a given *q* is mostly unrelated to the peaks in the transverse one at that same value of *q*.

**Figure 11:**
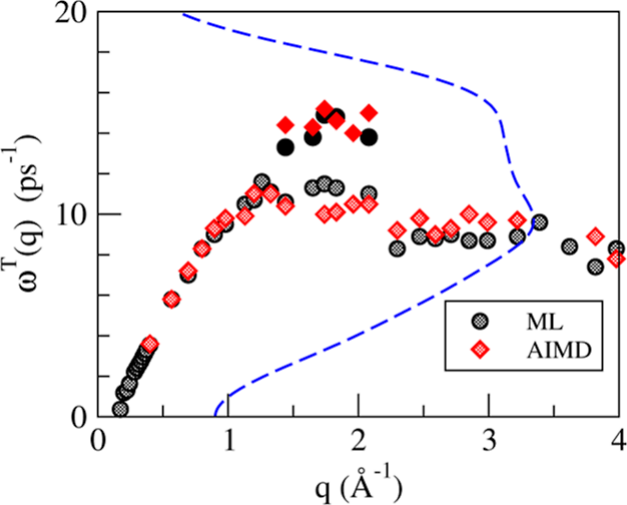
Transverse dispersion relation of l-La, as obtained from the maxima in the spectra of the transverse current, *C*^*T*^(*q*, ω). The blue dashed line represents the corresponding *Z*(ω).

The high- and low-frequency dispersion relations for the transverse modes are plotted in Figure 11. Some proximity is observed between the values of the high-frequency transverse peak, which is almost nondispersive, and the maximum in the longitudinal dispersion relation, as shown in Figure 8, which is located around *q*_p_/2. Such behavior has been previously observed in other systems^51−53^ and explained in terms of mode-coupling theories. This type of theories describes an indirect coupling between the transverse current at the wavevector considered, *q*, and the longitudinal magnitudes at all wavevectors, *k*, each one affected by a *q* and *k* (and also |*q* – *k*|)-dependent weight.^41,52,53^ It turns out that the maximum weight is obtained for *k* around *q*_p_/2 and *q* roughly in the range between *q*_p_/2 and *q*_p_, leading to the behavior observed in Figure 11.

Recently, it has been observed^53−60^ that some liquid metals exhibit the simultaneous appearance of such high-frequency transverse branch and the presence of a marked high-frequency peak/shoulder in the spectra of its velocity autocorrelation function, namely, *Z*(ω). The latter is defined as the FT into the frequency domain of , where the index 1 refers to a tagged ion in the fluid and ⟨····⟩ stands for the ensemble average. As shown in Figure 11, the present results for l-La confirm this connection between the structure of the *Z*(ω) and the existence of one or two transverse dispersion branches. There is not a complete theory that explains the common absence or presence of a high-frequency peak in *Z*(ω) and in *C*^*T*^(*q*, ω) for a certain *q*-range, especially because in case the peak exists in *Z*(ω), it is naturally assigned to the influence of longitudinal modes.^61^ Nevertheless, the possible existence of such a correlation has witnessed increasing computational evidence, and the present results for l-La engross the list of systems, where both features appear together.

The self-diffusion coefficient, *D*, can be determined from the time integral of *Z*(*t*), which has led to a value of *D* = 0.22 ± 0.01 Å^2^/ps. There are no experimental self-diffusion data for l-La; nevertheless, Iida and Guthrie^62^ inferred a value *D* = 0.34 Å^2^/ps, derived from some semiempirical expressions based on modified Stokes–Einstein-type formulas which relate the self-diffusion coefficient to other thermophysical magnitudes such as the density and the viscosity.

From the calculated *C*^*T*^(*q*, *t*), we also estimated the shear viscosity coefficient η, as follows.^41^ The memory function representation of *C*^*T*^(*q*, *t*), namely
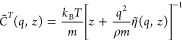
7where the tilde denotes the Laplace transform and introduces a generalized shear viscosity coefficient η̃(*q*, *z*). The area under the normalized *C*^*T*^(*q*, *t*) gives , and the extrapolation *q* → 0 of η̃(*q*, *z* = 0) ≡ η(*q*) gives the shear viscosity coefficient η. This procedure has been performed by fitting the ML simulation results with the expression, η(*q*) = η/(1 + *a*^2^*q*^2^), which was first proposed to analyze the results for a dense hard-sphere system^1^ but has also been applied to more complex systems.^63^

**Figure 12:**
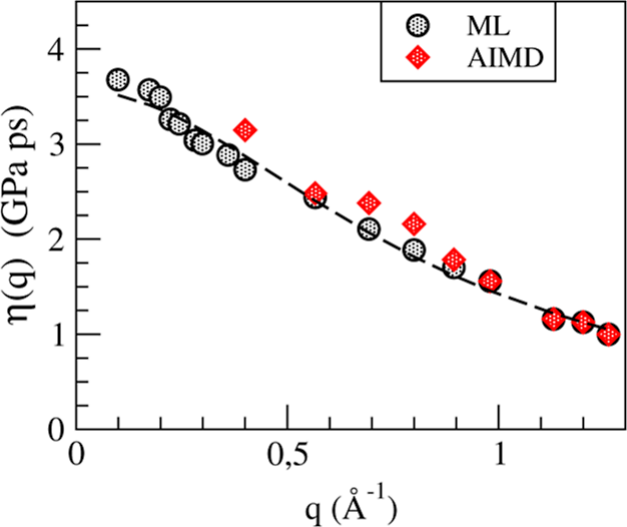
*q*-dependent shear viscosity, η(*q*) of l-La. The dashed line represents the fitting to the expression η(*q*) = η/(1 + *a*^2^*q*^2^).

Figure 12 shows the AIMD and ML simulation results for η(*q*) with the ML ones delving deeper into the small *q*-region. We have evaluated its long-wavelength limit, yielding a value for the shear viscosity, η = 3.40 ± 0.25 (GPa ps) which is close to one of the two reported experimental data η = 2.65, 3.27.^9^

#### 3.2.2 Liquid Li_4_Pb Alloy

For a two-component system, the AL partial intermediate scattering functions, *F*_*ij*_(*q*, *t*), are defined as a straightforward generalization of the definition previously given for a one-component system. Along with the BT partial intermediate scattering functions, they provide information about the collective dynamics of the fluctuations in the partial number densities, componentwise in the case of the AL functions, and topological and chemical in the case of the BT ones. Their time FT yields the respective AL and BT partial dynamic structure factors, *S*_*ij*_(*q*, ω) (see refs (64 and 65) for more details).

Another interesting magnitude is the *i*-type component particle current
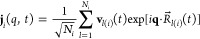
8because by evaluating the autocorrelation functions of its longitudinal, **j**_*i*_^*L*^(*q*,*t*), and transverse **j**_*i*_^*T*^(*q*, *t*) components, we obtain the partial longitudinal *C*_*ij*_^*L*^(*q*, *t*), and transverse, *C*_*ij*_^*T*^(*q*, *t*), current correlation functions, respectively.^64,65^ Moreover, it can be shown that the partial dynamic structure factors are related to the spectra of the partial longitudinal current correlation functions (both AL and BT), *C*_*ij*_^*L*^(*q*, ω), through the equation *C*_*ij*_^*L*^(*q*, ω) = (ω^2^/*q*^2^)*S*_*ij*_(*q*, ω).

All of the abovementioned partial correlation functions have been evaluated by both AIMD and ML simulations, and below we report the more interesting results as yielded by the ML simulations.

**Figure 13:**
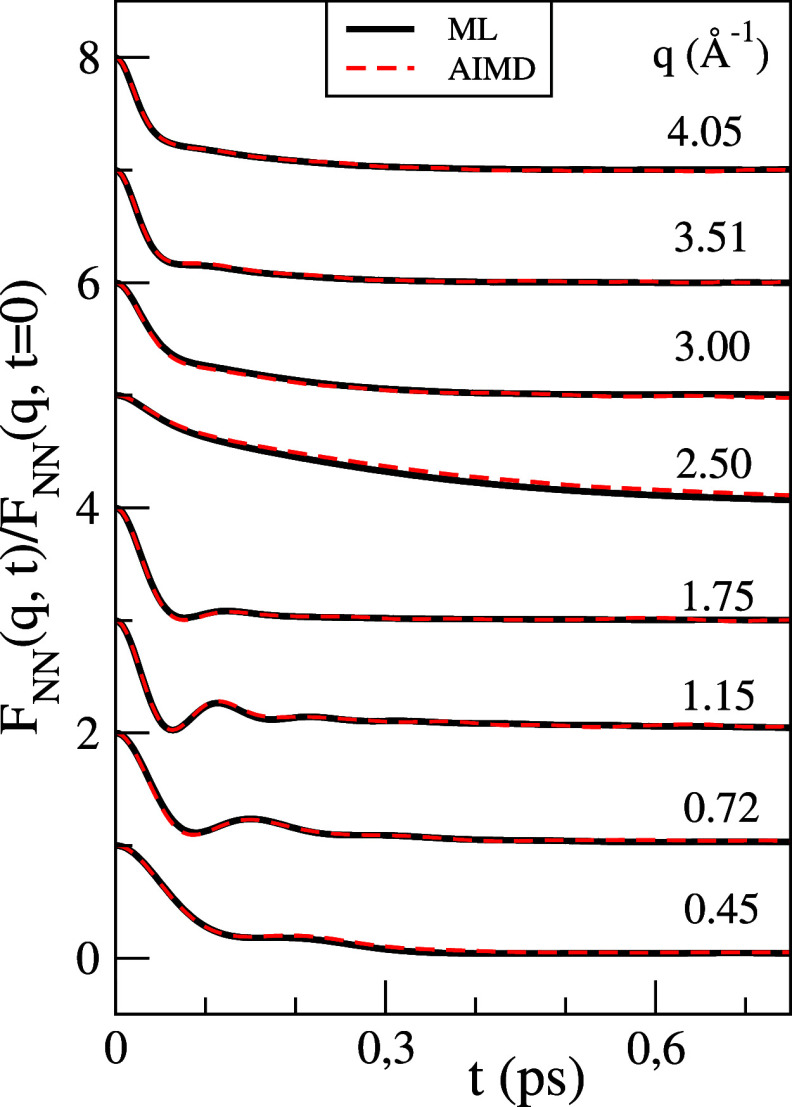
Normalized number–number intermediate scattering function, *F*_NN_(*q*, *t*)/*F*_NN_(*q*, *t* = 0), in the liquid Li_4_Pb alloy for several *q* values.

Figure 13 shows, for several *q*-values, the AIMD and ML results for the number–number intermediate scattering function, *F*_NN_(*q*, *t*). This function represents the autocorrelation function of the total number density in the alloy, and therefore, its behavior is qualitatively similar to the intermediate scattering function in a one-component system; namely, it has an oscillatory structure at small *q*-values which is depleted by the effect of a decaying contribution.

**Figure 14:**
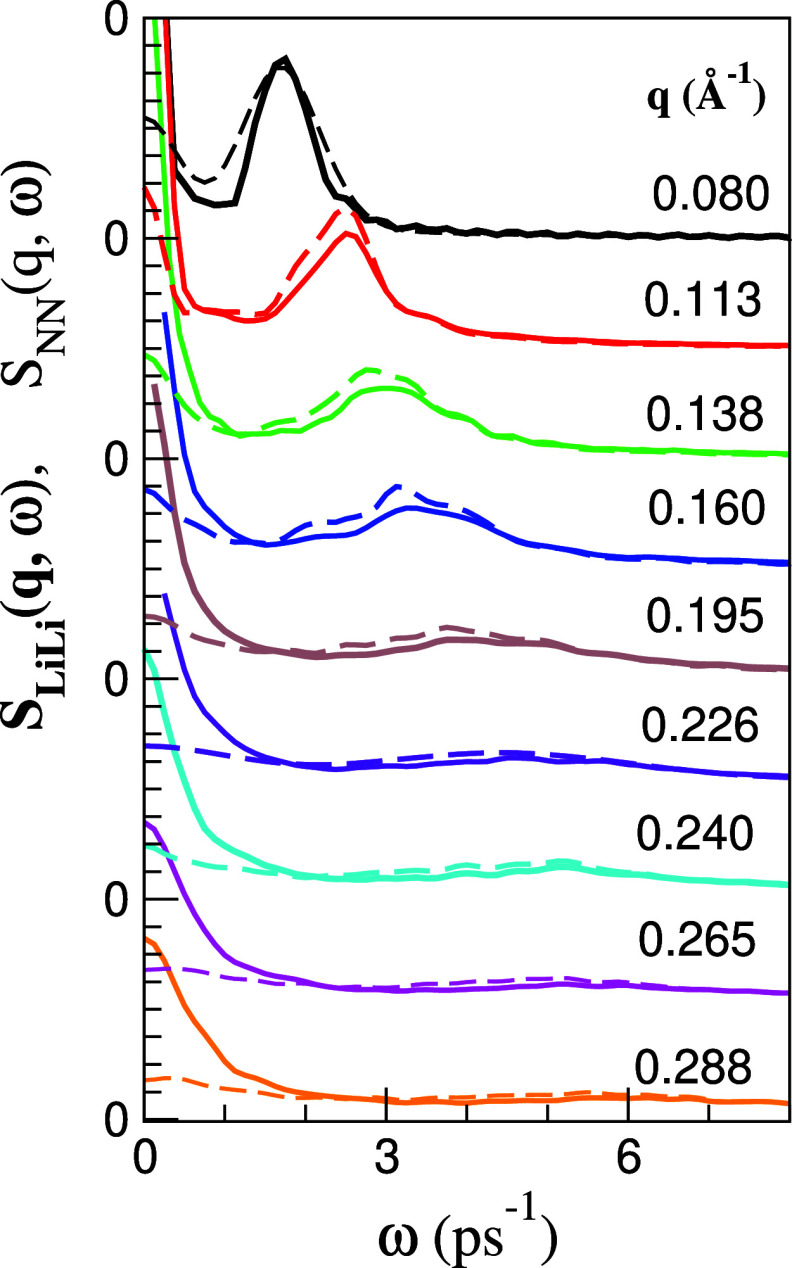
ML simulation results for the partial dynamic structure factors, *S*_LiLi_(*q*, ω), (full lines) and *S*_NN_(*q*, ω), (broken lines) in the liquid Li_4_Pb alloy for several small *q*-values.

The existence of propagating density fluctuations can be revealed by the appearance, within some *q*-range, of side peaks in the partial dynamic structure factors *S*_*ij*_(*q*, ω). Figure 14 shows, for the set of smallest attainable *q*-values, the obtained ML results for *S*_LiLi_(*q*, ω) and *S*_NN_(*q*, ω). The peaks are clearly visible, and with increasing *q*-values, its amplitude diminishes, and its position moves to higher frequencies.

**Figure 15:**
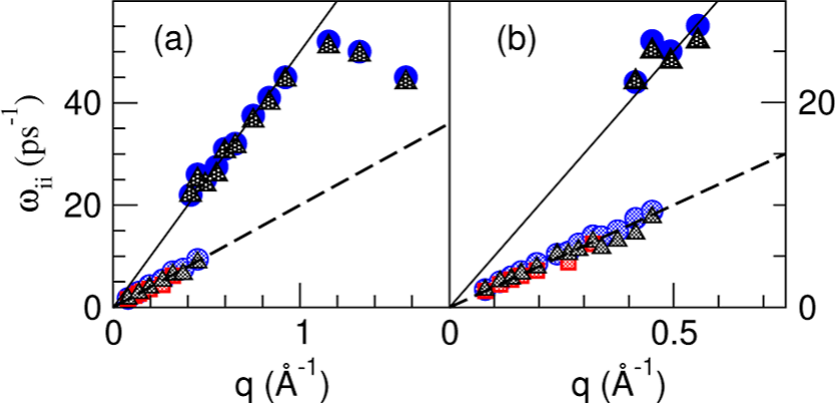
ML simulation results for the dispersion relations ω_LiLi_(*q*) (blue circles), ω_PbPb_(*q*) (red squares), and ω_NN_(*q*) (gray triangles) of the maxima in the partials *S*_LiLi_(*q*, ω), *S*_PbPb_(*q*, ω), and *S*_NN_(*q*, ω) for the Li_4_Pb liquid alloy. The slope of the dashed line corresponds to the experimental adiabatic sound velocity, whereas that of the full line corresponds to a phase velocity of 5000 m/s. (b) Closer insights into the low wavevector region.

From the frequencies associated with the positions of the side peaks, we have derived the corresponding dispersion curves, ω_LiLi_(*q*), ω_PbPb_(*q*), and ω_NN_(*q*), which are plotted in Figure 15. Notice that the ω_LiLi_(*q*) and ω_NN_(*q*) show two branches (high- and low-frequency branches), whereas the ω_PbPb_(*q*) displays one, low-frequency, branch. The low-frequency ω_LiLi_(*q*) branch appears for a range of *q* ≤ 0.45 Å^–1^ and basically coincides with the ω_PbPb_(*q*) and ω_NN_(*q*) branches. All of them, when *q* → 0 smoothly merge into a hydrodynamic sound. On the other hand, the high-frequency branch of ω_LiLi_(*q*) begins at around the same *q*-value where the low frequency one vanishes and, in principle, would be compatible with a phase velocity of ≈5000 m/s which is similar to the value associated to the high-frequency mode revealed in the INS data of Alvarez et al.^20^ Our results for the high-frequency branch of ω_LiLi_(*q*) show its appearance at *q* ≈ 0.45 Å^–1^ but do not allow us to conclude anything about its existence for smaller *q* values because it does not show up due to a small weight and/or, additionally, it is shielded by other, diffusive, modes.

The adiabatic sound velocity in the alloy, *c*_s_, has been obtained from the slope of ω_NN_(*q* → 0) with a value *c*_s_ ≈ 2030 ± 150 m/s which agrees very well with the experimental data of ≈2000 m/s.^66^ From the previous value for *c*_s_, along with the formula , where ρ_m_ is the mass density of the alloy, we have evaluated the adiabatic compressibility κ_ad_ in the alloy, obtaining a value κ_ad_ = 7.50 ± 0.20 (in units of 10^11^ m^2^ N^–1^) which is similar to the estimates suggested by Ruppersberg and Speicher.^67^ Moreover, taking into account that κ_*T*_ = γ_0_κ_ad_, where γ_0_ stands for the ratio of specific heats, and using the previous value for κ_*T*_, we obtain an estimate γ_0_ ≈ 1.09 (±0.05) which compares well with the theoretical value of γ_0_ ≈ 1.10 obtained by Bryk and Mryglod^17^ in their GCM study of this alloy.

In order to gather additional information about the previous collective excitation modes, we calculated the spectra of the longitudinal current correlation functions, *C*_*ij*_^*L*^(*q*, ω). These functions allow the exposure of longitudinal modes that are not visible in the *S*_*ij*_(*q*, ω) because the appearance of the factor ω^2^ attenuates the low-frequency modes (namely, the diffusive ones) and enhances the high frequency ones.

**Figure 16:**
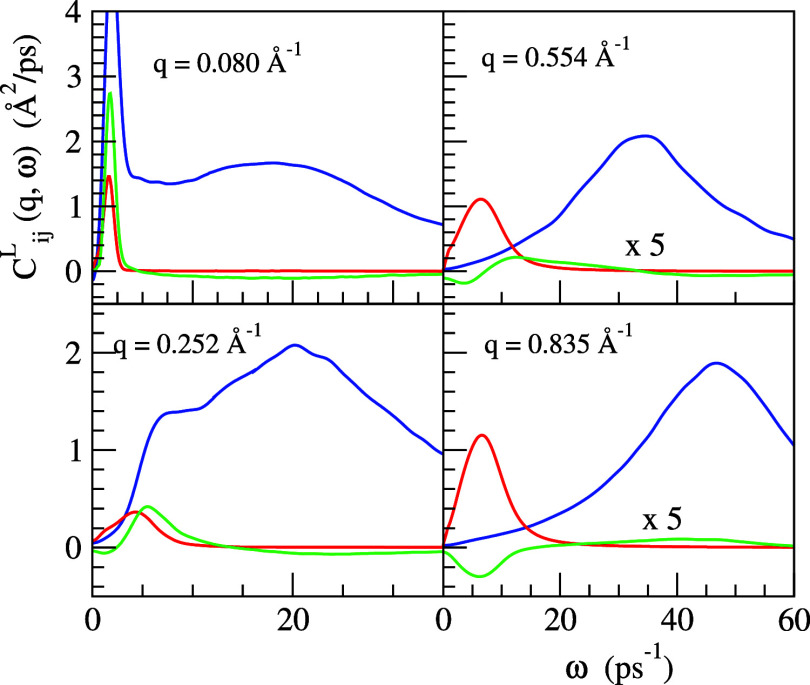
ML simulation results for the partial longitudinal current correlation functions, *C*_*ij*_^*L*^(*q*, ω), in the liquid Li_4_Pb alloy. Full blue line: *C*_LiLi_^*L*^(*q*, ω), red line: *C*_PbPb_^*L*^(*q*, ω), and green line: *C*_LiPb_^*L*^(*q*, ω). The PbPb and LiPb components for *q* = 0.554 and 0.835 Å^–1^ have been multiplied by a factor of 5 to ease comparison.

Figure 16 shows the ML calculated *C*_*ij*_^*L*^(*q*, ω) values for several small-*q* values. For *q* ≤ 0.26 Å^–1^, the *C*_PbPb_^*L*^(*q*, ω) displays just one peak, whereas the *C*_LiLi_^*L*^(*q*, ω) and *C*_NN_^*L*^(*q*, ω) exhibit two peaks. At greater *q*’s, all just show one peak, which is the high frequency one in the case of the LiLi and NN functions. On the other hand, *C*_LiPb_^*L*^(*q*, ω) has extrema that can be either positive or negative. Interestingly, in the region *q* ≤ 0.26 Å^–1^, the positive peak in *C*_LiPb_^*L*^(*q*, ω) coincides in the position with the peaks in *C*_LiLi_^*L*^(*q*, ω) and *C*_PbPb_^*L*^(*q*, ω), which suggests the existence of an acoustic mode, where all the atoms/ions vibrate in phase.

**Figure 17:**
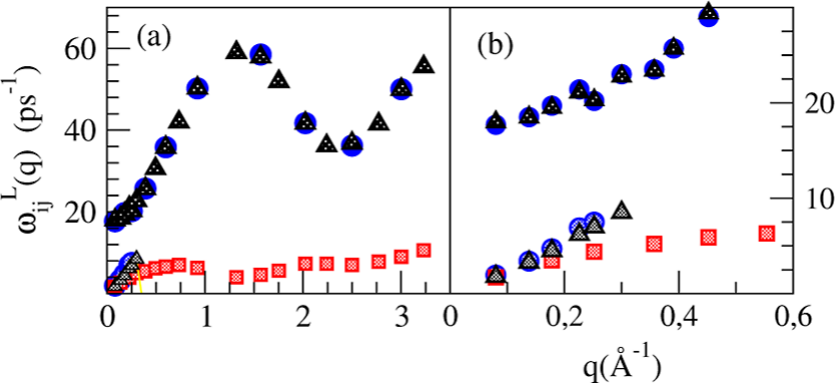
(a) ML simulation results for the longitudinal dispersion relations ω_LiLi_^*L*^(*q*) (blue circles), ω_PbPb_^*L*^(*q*) (red squares), and ω_NN_^*L*^(*q*) (gray triangles) for the Li_4_Pb liquid alloy. (b) Closer insights of the low wavevector region.

From the positions of the peaks in *C*_*ij*_^*L*^(*q*, ω), we have obtained the longitudinal dispersion relations, ω_*ij*_^*L*^(*q*), which are plotted in Figure 17. There appear two branches for ω_LiLi_^*L*^(*q*) and ω_NN_^*L*^(*q*) and one branch for ω_PbPb_^*L*^(*q*). From the ML results, we can get full insights into the small *q* behavior of the high-frequency branch of ω_LiLi_^*L*^(*q*) which clearly tends to a finite value when *q* → 0; this result clearly shows the kinetic (nonhydrodynamic) nature of this excitation and discards previous claims concerning the fast sound character of this mode.

**Figure 18:**
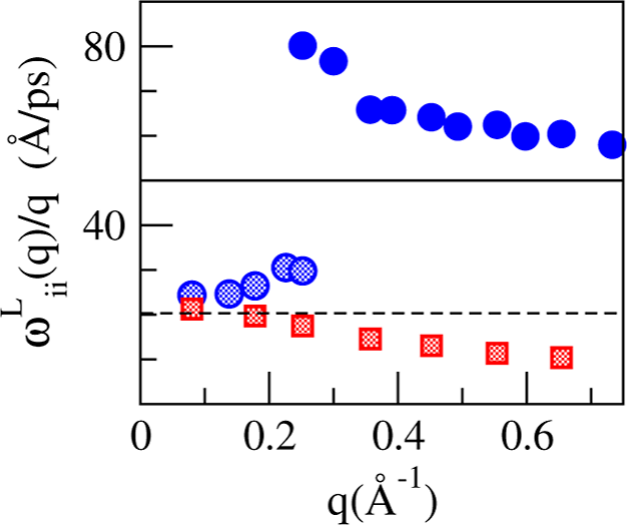
Phase velocities, ω(*q*)/*q* corresponding to the dispersion relations of the longitudinal currents. Symbols are the same as in the previous figure, and lines correspond to the hydrodynamic sound velocity and the fast sound velocity deduced from the peak positions of *S*_LiLi_(*q*, ω).

In order to make connection with the previous simulations that interpreted a merging of fast and slow sound into the hydrodynamic sound mode as *q* → 0,^16^ we have calculated the phase velocities corresponding to the dispersions of the longitudinal currents, which are plotted in Figure 18.

As in ref (16), we observe a merging of two phase velocities into the hydrodynamic *c*_s_, one from below (corresponding to Pb) and the other one from above (corresponding to Li). However, it is clear that the higher one cannot be identified with the fast sound mode. The fast sound phase velocity, when *q* → 0, does not bend down abruptly to match the one that goes toward *c*_s_ from above as inferred in ref (16). On the contrary, it increases toward infinity as a consequence of the optical character of the mode with a nonzero frequency at *q* = 0.

We have also evaluated the partial transverse current correlation functions as they provide information about the shear modes in the alloy and its viscosity. An important magnitude in the following discussion is the total transverse current correlation function *C*_tt_^*T*^(*q*, *t*) = ⟨*j*_*t*_^*T*^(*q*, *t*)*j*_*t*_^T*^(*q*, 0)⟩, where
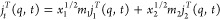
9is the total transverse current, *m*_*i*_ (*i* = 1, 2) is the atomic mass of component *i*, and *j*_*i*_^*T*^(*q*, *t*) is the transverse component of the current defined in (8).

**Figure 19:**
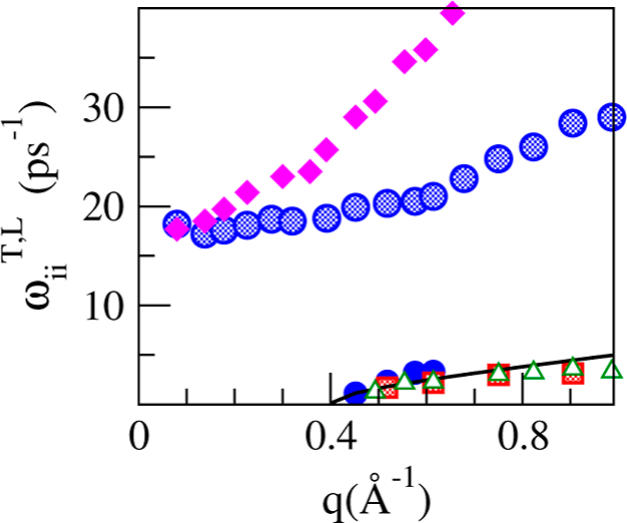
ML simulation results for the transverse dispersion relations ω_LiLi_^*T*^(*q*) (full and shaded blue circles), ω_PbPb_^*T*^(*q*) (shaded red squares), and ω_tt_^*T*^(*q*) (open green triangles), for the Li_4_Pb liquid alloy. The magenta diamonds correspond to the high-frequency longitudinal ω_LiLi_^*L*^(*q*). The continuous line represents the data obtained from eq 10.

The *C*_PbPb_^*T*^(*q*,ω) and *C*_tt_^*T*^(*q*,ω) are found to have just a low-frequency peak, whereas *C*_LiLi_^*T*^(*q*,ω) has a high-frequency peak and, for a limited *q* range, also another low-frequency one. The corresponding transverse dispersion relations, ω_*ii*_^*T*^(*q*), are shown in Figure 19.

The low-frequency transverse excitations correspond to shear waves that appear after a finite propagation gap. Near the edge of the propagation gap, the frequencies of the shear waves can be reasonably described by a viscoelastic model,^68^ with
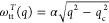
10where *q*_c_ is the minimum wavevector for the existence of shear waves which in the present calculation gave the value *q*_c_ = 0.401 Å^–1^. For *q* values somewhat larger than *q*_c_ the low-frequency branch can also be described by a linear form, ω_tt_^*T*^(*q*) ∼ *c*_*T*_(*q* – *q*_*T*_), where the slope, *c*_*T*_, yields an estimate of the velocity of propagation of the shear modes in the alloy, *c*_*T*_ ≈ 1250 ± 150 m/s.

The high-frequency branch of ω_LiLi_^*T*^(*q*) tends to a finite nonzero frequency in the long wavelength limit. This value is observed to coincide with the value taken by the longitudinal high-frequency branch, also shown in Figure 19, in this limit. This is the expected behavior for the optical longitudinal and transverse modes of a non-ionic binary liquid. Should unscreened Coulombic interactions between ions exist, then a gap between the longitudinal optic and transverse optic modes at *q* → 0 would appear.^68^

We finally report the alloy shear viscosity, which has been evaluated by a method that is just an extension of the one-component formulation (see eq 6) to the case of binary systems, using the total transverse current correlation function, *C*_tt_^*T*^(*q*,*t*), defined previously (for more details, consult refs (69 and 70)). By resorting to the memory function representation of *C*_tt_^*T*^(*q*, *t*), we obtain a generalized shear viscosity coefficient, η̃(*q*, *z*), and the extrapolation to *q* → 0 of η(*q*) ≡ η̃(*q*, *z* = 0) gives the alloy shear viscosity.

**Figure 20:**
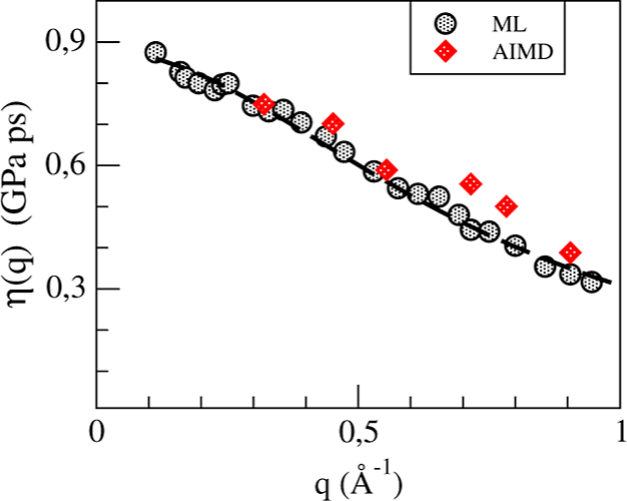
*q*-dependent shear viscosity, η(*q*) of the liquid Li_4_Pb alloy. The dashed line represents the fitting to the expression mentioned in the text.

Figure 20 shows the AIMD and ML results for η(*q*). The *q* → 0 limit of the ML results yields a value for the shear viscosity of η = 0.89 ± 0.05 (GPa ps). We are unaware of any experimental data to compare with, but there is some experimental trend according to which if an alloy exhibits heterocoordinating tendencies, as is the case in this alloy, then its shear viscosity usually shows a positive deviation from linearity. From the experimental values of the pure components at *T* = 1075 K,^9^ i.e., η(Li) = 0.27 ± 0.05 and η(Pb) = 1.22 ± 0.10 (GPa ps), a linear variation gives a value that is half the present result. Therefore, a positive deviation from linearity clearly occurs, as might be expected from the strong heterocoordinating tendency of Li_4_Pb.

## 4 Conclusions

We have applied the ML method to develop accurate interatomic potentials for both l-La and the Li_4_Pb alloy. These potentials were trained on AIMD simulations and tested by comparing a range of static, dynamics, and transport properties with those previously calculated by the AIMD simulations.

The results reported for l-La constitute the first AIMD study of its static and dynamic properties, with previous studies employing only semiempirical models. We analyzed the static structure through the pair distribution function, *g*(*r*), and the static structure factor, *S*(*q*). We identified an asymmetric shape in the second peak in *S*(*q*) with a marked shoulder. This feature has been linked to the existence of icosahedral short-range order in the liquid. A further calculation using the CNA method revealed a significant presence of perfect and distorted icosahedral structures, consistent with previous results linking the icosahedral structure to an asymmetric second peak in *S*(*q*).

In addition, we have determined the generalized specific heat ratio, γ(*q*) by analyzing the relaxation processes behind the intermediate scattering function. The ML simulation results have proven to be crucial in revealing the low-*q* shape of γ(*q*) which appears qualitatively similar to the experimental γ(*q*) of l-Fe, as found by Hosokawa.^49^ Although no direct experimental data are available, the extrapolated value γ(*q* → 0) ≡ γ_0_ agrees with some semiempirical estimates.

The calculated dynamic structure factors, *S*(*q*, ω), show side peaks that are indicative of collective density excitations. According to ML results, the associated dispersion relation shows the existence of a positive dispersion that could not be detected within the AIMD calculations. The transverse dispersion relation for l-La exhibit two branches consistent with the suggested connection between the structure of the spectra of the VACF and the existence of one/two transverse dispersion branches. Results have also been reported for several transport coefficients, namely, the self-diffusion, adiabatic sound velocity, and shear viscosity coefficients. Taking into account the scarcity of data for most of these transport coefficients, we expect that the present results will be of interest for future experimental and theoretical research.

Despite all the previous work on the liquid Li_4_Pb alloy, there remained some controversial points about its properties, in particular about the nature of the high-frequency excitation associated with the LiLi dispersion relation, that have been addressed in detail in the present study. The reported ML simulations have delivered results for its static and dynamic properties with a precision matching the AIMD simulation results. However, the capability of the ML simulations to provide accurate information about the *q* → 0 behavior of any *q*-dependent physical magnitude has been key to perform a very detailed investigation of the high-frequency excitation branch related to the LiLi dispersion relation. The ML simulation results show that this excitation is a kinetic (nonhydrodynamic) optic mode, and given the near ab initio precision of the present ML simulations, we can confidently rule out the fast sound character of this excitation. We have moreover reconciled the different views on the behavior of this alloy, showing that indeed, despite the kinetic optical nature of the high-frequency “fast sound” mode, a merging does exist between the phase velocities associated with the low-frequency peaks of the longitudinal currents associated with the heavy and light particles.

Overall, we have shown the power of ML interatomic potentials to analyze with higher accuracy multiple properties while, at the same time, finally discern the origin of some special features discovered in recent years.
